# Two novel members of Onygenales, *Keratinophyton kautmanovae* and *K. keniense* spp. nov. from soil

**DOI:** 10.1038/s41598-024-67475-y

**Published:** 2024-07-17

**Authors:** Roman Labuda, Vanessa Scheffenacker, Andreas Schüller, Broňa Voleková, Alena Kubátová, Hazal Kandemir, Winnie Cherotich Maritim, Josphat Matasyoh, Markus Gorfer, Christoph Schüller, Joseph Strauss

**Affiliations:** 1 Department for Farm Animals and Veterinary Public Health, Institute of Food Safety, University of Veterinary Medicine Vienna (VetMed), Veterinaerplatz 1, 1210 Vienna, Austria; 2 Research Platform Bioactive Microbial Metabolites (BiMM), Konrad Lorenz Strasse 24, 3430 Tulln an der Donau, Austria; 3 Core Facility Bioactive Molecules Screening and Analysis, Konrad Lorenz Strasse 24, 3430 Tulln an der Donau, Austria; 4 Department of Applied Genetics and Cell Biology, Institute of Microbial Genetics, University of Natural Resources and Life Sciences, Vienna (BOKU), Campus Tulln, Konrad Lorenz Strasse 24, 3430 Tulln an der Donau, Austria; 5 Slovak National Museum-Natural History Museum, Vajanského náb. 2, P. O. Box 13, 810 06 Bratislava, Slovak Republic; 6 Department of Botany, Culture Collection of Fungi (CCF), Faculty of Science, Charles University, Benátská 2, 128 00 Prague 2, Czech Republic; 7 Westerdijk Fungal Biodiversity Institute, Uppsalalaan 8, 3584 CT Utrecht, The Netherlands; 8 Department of Chemistry, Faculty of Sciences, Egerton University, P.O. Box 536, 20115 Egerton, Kenya; 9 Bioresources, Center for Health and Bioresources, AIT Austrian Institute of Technology GmbH, Konrad-Lorenz-Straße 24, 3430 Tulln an der Dona, Austria

**Keywords:** *Chrysosporium* asexual morph, Hair baiting method, Keratinophilic fungi, New taxa, Evolution, Microbiology, Molecular biology, Systems biology

## Abstract

Two new *Keratinophyton* species, *K. kautmanovae* sp. nov. and *K. keniense* sp. nov., isolated from soil samples originating from two different geographical and environmental locations (Africa and Europe) are described and illustrated. Phylogenetically informative sequences obtained from the internal transcribed spacer (ITS) region and the nuclear large subunit (LSU) rDNA, as well as their unique phenotype, fully support novelty of these two fungi for this genus. Based on ITS and LSU combined phylogeny, both taxa are resolved in a cluster with eight accepted species, including *K*. *alvearium*, *K*. *chongqingense*, *K*. *hubeiense*, *K. durum*, *K. lemmensii*, *K*. *siglerae*, *K. submersum*, and *K. sichuanense*. The new taxon, *K. kautmanovae*, is characterized by clavate, smooth to coarsely verrucose conidia, absence of arthroconidia, slow growth at 25 °C, and no growth at 30 °C, while *K. keniense* is morphologically unique with a high diversity of conidial shapes (clavate, filiform, globose, cymbiform and rhomboid). Both species are described based on their asexual, a chrysosporium-like morph. While the majority of hitherto described *Keratinophyton* taxa came from Europe, India and China, the new species *K. keniense* represents the first reported taxonomic novelty for this genus from Africa.

## Introduction

The genus *Keratinophyton* was introduced in 1964 by Randhawa & Sandhu^1^ However, the nomenclatural history of this genus was confusing since untill recently all known *Keratinophyton* species were classified in *Aphanoascus* (Cooke) Apinis^2–4^. Members of *Keratinophyton* and *Aphanoascus* share cleistothecia with a pseudoparenchymatic peridium, and they can be found on keratinous substrata and dung^3,5,6^. Sutton et al. (2013) re-established the genus *Keratinophyton* with *K. terreum* as the type species, based on ascospore morphology^5^. These two genera are distinguished by their ascospore morphology, where ascospores with a conspicuous equatorial rim and pitted wall are characteristic for *Keratinophyton*, while *Aphanoascus* species produce reticulate ascospores without a rim^5^, and by result of phylogenetic analysis using ITS (the internal transcribed spacer) and LSU [the D1/D2 domains of the large-subunit (28S) rDNA gene] loci^5–7^. The monophyletic *Keratinophyton* clade currently encompasses six species, namely *K. durum*, *K. hispanicum*, *K. multiporum*, *K. punsolae*, *K. saturnoideum*, and *K. terreum*, known to form in vitro ascosporic state (sexual morph) as well as 19 taxa known only as asexual morphs, characterized mainly by a chrysosporium-like conidiogenesis^6–8^. Actually, the genus *Keratinophyton* includes several species described in *Chrysosporium*^9–14^. These species however, have been regarded as having a doubtful identity^6^.

The genus *Keratinophyton* is represented by a large group of keratinolytic soil-borne fungi rather common in areas with high animal activity resulting in transfer of keratinous material to the soil. Ecology and distribution of the genus has been reviewed in a previous study, stating that soil and soil-like substrata are primary habitats for this fascinating group of onygenalean fungi^7^. Currently, this genus comprises a total of 27 recognized and accepted taxa^6,8^.

We performed a mycological survey with emphasis on keratinophilic fungi in environmental samples taken from Republic of Kenya, Africa and Slovak Republic, Europe. Herein, we present description of two novel morphologically and phylogenetically distant species within the genus *Keratinophyton* being characterized by their unique chrysosporium-like morphs.

## Material and methods

### Sample collection and isolation of the fungi

The soil sample was collected in front of the Budúcnosť adit at the abandoned antimony (Sb) deposit Pezinok—Kolársky Vrch, 48°19′03.3" N 17°14′20.9" E, locality Rudné Mines, Pezinok (Slovak Republic) in December 2021. Sampling site was situated approximately one meter from neutral mine drainage contaminated by potentially toxic elements.

The second soil sample was collected from Egerton University campus, 0°22′09.3" S 35°55′24.2" E, Njoro (Republic of Kenya) in February 2022.

The samples from the surface layer (up to 20 cm deep) were dried and stored in at 5‒8 °C. Isolation of the keratinophilic fungi was performed as previously described^15^, with a modification according to^7^. A sample was divided into five subsamples. The subsamples (5 g each) were poured into Petri dishes (50 mm in diameter) and mixed with 0.5 g Vermiculite, then soaked with 3‒4 mL (depending on moiety of the sample) antibiotic solution containing 0.5 g/L cycloheximide and 0.1 g/L chloramphenicol. Sterile defatted horse hair fragments (10 pieces of ca 2.0 cm per plate) were used as baits. The Petri dishes were then incubated at laboratory temperature (23‒25 ± 1 °C), in dark, for a period of 2–3 months and remoistened with sterile deionized water when necessary^7,15^. The Petri dishes were checked weekly for the presence of fungi, and colonies were transferred on Sabouraud 4% dextrose agar (SDA; Merck, Darmstadt, Germany) supplemented with 0.5 g cycloheximide and 0.05 g chloramphenicol. Pure cultures were then transferred onto potato dextrose agar (PDA; Van Waters and Rogers (VWR) International, Leuven, Belgium)^7^.

### Morphological analysis

The preliminary identification of the resulting keratinophilic fungi was carried out based on their phenotypic characteristics^9–11^.

For phenotypic determination, the strains were transferred by three-point inoculation onto PDA, Malt Extract Agar (MEA; Merck, Darmstadt, Germany), and SDA, and incubated for 14 d in the dark at 25 °C. Christensen´s urea agar (Sigma-Aldrich, St Louis, MO, USA) was used for additional physiological and biochemical characteristics (25 °C, 14 days, in the dark)^7^.

Colony growth rate (mm), colony structure and characteristics such as production of exudates and pigments were noted after 14 days (on PDA, MEA, and SDA). However, the cultivation was extended up to 3 months to observe and record changes in pigmentation of the colonies as well as to determine the onset of sexual reproduction^7^. In order to determine the optimal and minimum/maximum temperatures for growth, PDA, MEA and SDA plates were incubated at 5 °C, 8 °C, 10 °C, 12 °C, 15 °C, 18 °C, 20 °C, 25 °C, 28‒32 °C, 35 °C, and 37 °C, and the growth rate was measured on the 14th day of cultivation. For comparative descriptions of the macroscopic and microscopic characteristics, PDA was used according to^7,11,16^.

For observation of microscopic traits 14‒18 days growth on PDA was used. Conidiophore and conidia formation were observed in situ under low magnification (50–100×). Details of conidiophores, conidia (aleurioconidia) and other microscopic structures, such as width of hyphae, were observed in Melzer´s reagent and lactic acid with cotton blue^7^. Photomicrographs were taken using phase and Nomarski contrast optics on an Olympus BX51 microscope with Olympus DP72 camera and QuickPHOTO Micro 3.0 software. Photographs of the colonies were taken with a Sony DSC-RX100.

Dried fungal specimens were deposited as holotypes in the collections of the Mycological Department, National Museum in Prague, Czech Republic (PRM); ex-type cultures were deposited in the Bioactive Microbial Metabolites (BiMM) Fungal Collection, UFT- Tulln in Austria and in the Culture Collection of Fungi in Prague (CCF)^7^.

### Keratinolytic activity

A hair perforation test was performed following de Hoog et al. (2020) using 25 mL water containing 2–3 drops 10% yeast extract (YEW)^17^. The hairs were examined microscopically 14 and 21 days after inoculation at 25 °C in the dark. At the end of the incubation period, a few pieces of hair were taken out from the testing medium. The overgrowing fungus was deactivated with 70% ethanol and then removed from the hair surface mechanically in a stream of a tap water^7^. The degree of hair digestion-degradation (keratinolytic activity) was assessed in the light microscope under 100× and 400× magnification. Water was used as mounting fluid for the observation and microphotography of the hair samples. Intensity of degradation^18^ of hair was estimated on a scale of 0 to 4: 0 = no degradation; 0–1 = light degradation on the cuticle; 1 = moderate degradation on the cuticle and/or rare formation of boring hyphae; 2 = degradation of cuticle and cortex, with about 20% degradation; 3 = degradation of cuticle and cortex, with about 50% degradation; 4 = degradation of cuticle and cortex, with about 80% degradation.

### DNA extraction, PCR amplification and sequencing

DNA was extracted using a standard cetyltrimethyl ammonium bromide (CTAB) procedure, as described previously^7,19^. The internal transcribed spacer (ITS) region was amplified with primers ITS1-F^20^ and ITS4^21^ using Taq-polymerase (GoTaq G2 Green Master Mix from Promega). The *D1*/*D2* domains of the large-subunit (28S) rDNA gene (LSU) were amplified and sequenced using the primer pair ITS1/TW14^21,22^. All reactions were performed in an Eppendorf Gradient *MasterCycler* (Eppendorf, Hamburg, Germany). Conditions for amplification of ITS and LSU domains: 95 °C for 5 min; 35 cycles of 95 °C for 30 s, 54 °C for 30 s, 72 °C for 90 s, and finally 5 min at 72 °C^7^. The PCR products were sequenced with the same primers used for the PCR amplifications (*LGC*, Berlin, Germany). All sequences obtained in this study were deposited in GenBank nucleotide database (Table 1).

**Table 1:** List of the strains included in the study.

Species name	Strain	Source	GenBank accession numbers	
			ITS	LSU
*A*.* canadensis*	UAMH 4574	Carnivore dung, Canada	AJ439435	–
*A*.* clathratus*	IMI 329400	Arable soil, Spain	AJ439436	–
*A*.* cubensis*	FMR 4220	Soil of tobacco field, Cuba	AJ439432	–
*A*.* foetidus*	CBS 453.75^T^	*Myomys daltoni* coat, Nigeria	KT155907	KT155252
*A. fulvescens*	NBRC 30411	Soil of rice paddy field, Japan	JN943432	JN941547
*A*.* keratinophilus*	IFM 55159^T^	Pasture land soil, Papua New Guinea	NR165936	NG064030
*A*.* mephitalis*	IMI 151084^T^	Dung of wolf, Canada	AJ439439	AY176725
*A*.* orissae*	CBS 340.89	Soil in animal husbandry, Kuwait	AJ390393	–
*A*.* pinarensis*	FMR 4221	Forest soil, Cuba	AJ439433	–
*A*.* reticulisporus*	CBS 392.67^T^	Soil, New Zealand	MH859002	MH870704
*A*.* verrucosus*	NBRC 32381^T^	Arable soil, Spain	NR131309	NG057011
*K. alvearium*	LC 11684^T^	Hive-stored pollen, China	MF939598	MF939580
	CGMCC 3.20866	Soil, China	OM952124	OM952112
*K*.* clavisporum*	G80.1^T^	Plant root soil, China	KY026601	–
*K. chongqingense*	CGMCC3.20867^T^	Green belt soil, China	OM952125	OM952113
	GZUIFR 22.030		OM952126	OM952114
*K*.* durum*	CBS 118.85^T^	Soil, Nepal	MH861856	AB075345
*K*.* echinulatum*	CCF 4652^T^	Sole of the foot, Czechia	LT548276	LT548276
*K*. *evolceanui*	CBS 116.63T	Soil, India	AJ005368	MH869834
*K*.* fluviale*	FMR 6005^T^	River sediments, Spain	AJ005367	MT875000
	CGMCC 3.20869	Soil, China	OM952132	OM952120
	GZUIFR 22.034	Soil, China	OM952133	OM952121
*K*.* gollerae*	BiMM F250^T^	Forest soil, Slovakia	MN633084	MT874997
*K*.* hispanicum*	CBS 456.90^T^	Beach soil, Spain	KT155910	MT875003
*K*.* hubeiense*	EM66601^T^	Soil under the chicken feather, China	KJ849227	–
	CGMCC 3.20870	Soil, China	OM952128	OM952116
*K. indicum*	CBS 117.63^T^	Soil, India	NR_145203	KT155044
***K. kautmanovae***	**BiMM F297**^**T**^ **BiMM 2296**	**Soil nearby a Sb stream Slovakia** **Soil nearby a Sb stream Slovakia**	**PP062810** **PP062811**	**PP062954** **PP062955**
***K. keniense***	**BiMM F335**^**T**^	**Soil, Republic of Kenya**	**PP062809**	**PP062953**
*K*.* lemmensii*	BiMM F76^T^	Compost soil, Austria	MN633082	MT874998
*K*.* linfenense*	GZAC H31^T^	Rhizosphere soil, China	NR158289	–
*K*.* minutisporosum*	IMI 379912^T^	River sediments, Spain	KT155616	MT875001
*K*.* punsolae*	IMI 334818^T^	Arable soil, Spain	AJ439440	–
*K*.* qinghaiense*	GZUIFR Chry 11^T^	Farmland soil, China	JX868607	–
	CGMCC 3.20872	Soil, China	OM952134	OM952122
*K*.* saturnoideum*	CBS 628.88^T^	Arable soil, Spain	NR077135	AB075347
*K. sichuanense*	CGMCC 3.20871^T^	Green belt soil, China	OM952130	OM952118
	GZUIFR 22.032	Green belt soil, China	OM952129	OM952117
*K*.* siglerae*	UAMH 6541^T^	Garden soil, Spain	AJ131684	MT875002
*Keratinophyton* sp.	CBS 503.63	Forest soil, India	KT155929	KT155274
*K*.* straussii*	BiMM F78^T^	Garden soil, Italy	MN633081	MT874996
*K*.* submersum*	CBS 101575^T^	River sediments, Spain	NR157445	NG064180
*K*.* terreum*	CBS 342.64^T^	Lawn soil, India	KT155876	KC989709
*K*. *turgidum*	CBS 142596^T^	Barber shop soil, India	KY290503	KY962732
*K*.* wagneri*	BiMM F77^T^	Forest soil, Slovakia	MN633083	MT874999
*Ct*. *serratus*	CBS 187.61^T^	Soil, Australia	NR144890	AY176733

Data in bold generated in the present study.

*BiMM* bioactive microbial metabolites unit, UFT-Tulln, Austria, *CBS* (Westerdijk Fungal Biodiversity Institute), Utrecht, The Netherlands, *CCF* culture collection of fungi, Charles University, Prague, Czech Republic, *EM, and GZUIFR = CGMCC = LC strains* The Institute of Fungus Resource, Guizhou University, China, *FMR* Facultat de Medicina in Ciències de la Salut, Reus, Spain, *IMI* CAB International Biosciences, Egham, UK, *NBRC* IFO, Institute for Fermentation, Osaka, Japan, *NITE* Biological Resource Centre, Japan, *UAMH* University of Alberta Microfungus Collection and Herbarium, *G*, *A*, *Aphanoascus,* *K* *Keratinophyton,* *Ct* *Ctenomyces*, ^*T*^ ex-type culture.

### Phylogenetic analysis

All sequences were aligned with MAFFT v7 with default settings. The percent similarity between strains was determined using BioEdit v7.2^23^. ModelFinder^24^ on IQ-TREE web server^25^ was used to find the best-fitting model for ITS and LSU datasets according to the Bayesian Information Criterion (BIC). Phylogenetic trees were constructed using the maximum likelihood (ML) methods implemented in IQ-TREE web server. Branch support values were measured using ultrafast bootstraps. Additionally, MrBayes v3.2.7^26^ with default settings on the CIPRES portal (http://www.phylo.org/) was used for both datasets. *Ctenomyces serratus* CBS 187.61 (*Arthrodermataceae*, *Onygenales*) was used as an outgroup. Phylogenetic trees were displayed and edited using Treeview v1.6.6^27^ and iTOL v6^28^.

## Results

### Morphological analyses and keratin degradation

The results of the morphological analyses are given for each novel species under the Taxonomy section below. Temperature dependent growth of the new *Keratinophyton* species on PDA, MEA and SDA after 14 days are provided in Table S1a‒c. Briefly, *K*. *keniense* grew better than *K. kautmanovae* on the same type of media and at the same incubation temperatures. All species showed good growth at 20‒25 °C on all three media. *K. kautmanovae* does not grow at 30 °C, while *K. keniense* reached up to 38 mm after 14 days at this temperature.

Ability to digest keratin was observed in the two new species after 21 days on testing medium (YEW). However, attack intensity on the hair according to the scale of^18^ Marchisio et al. was detected to be very weak in both species (= 0–1). Urease activity was negative in both new species on Christensen ´s urea agar.

### Phylogenetical analysis

**Figure 1:**
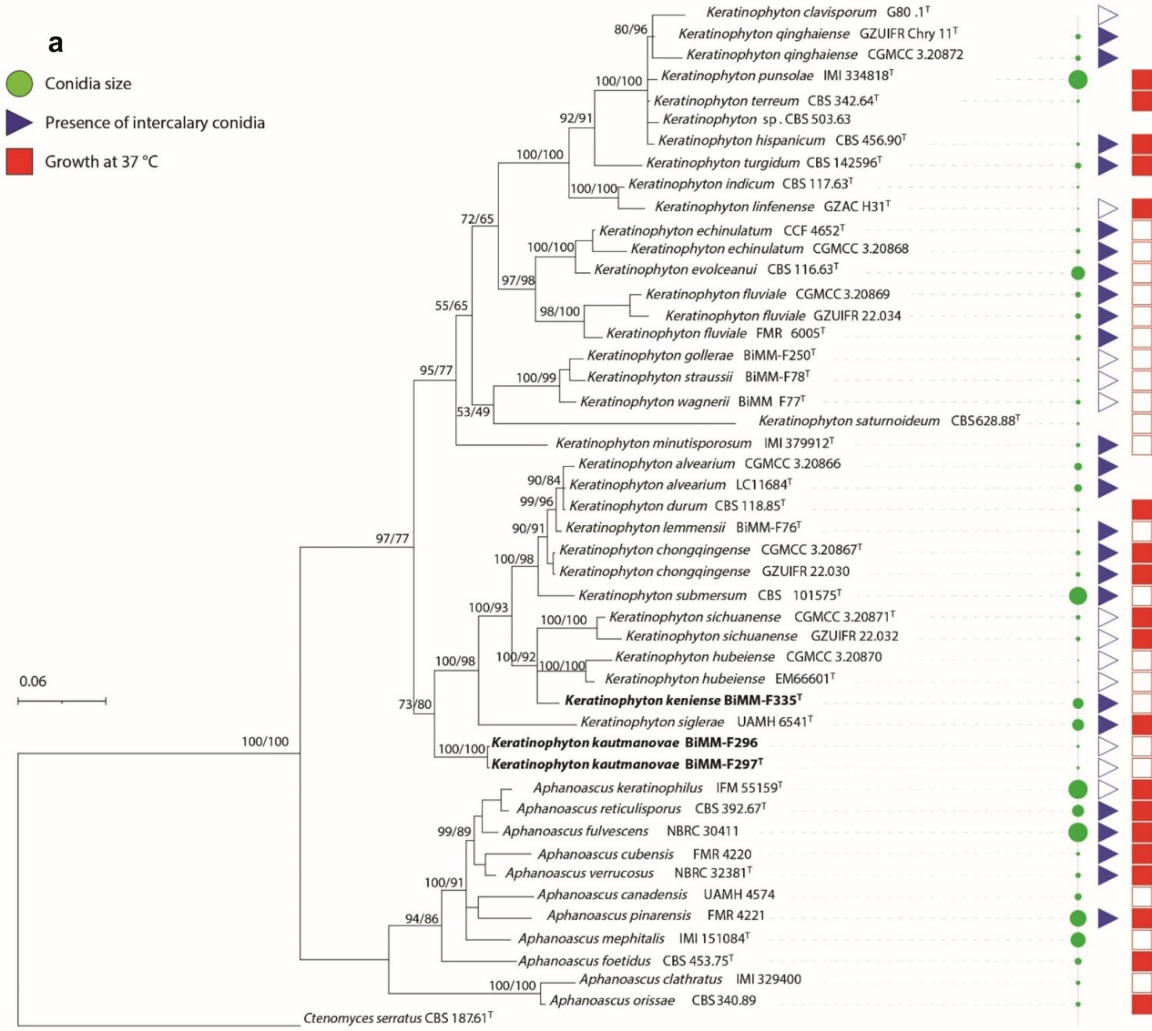

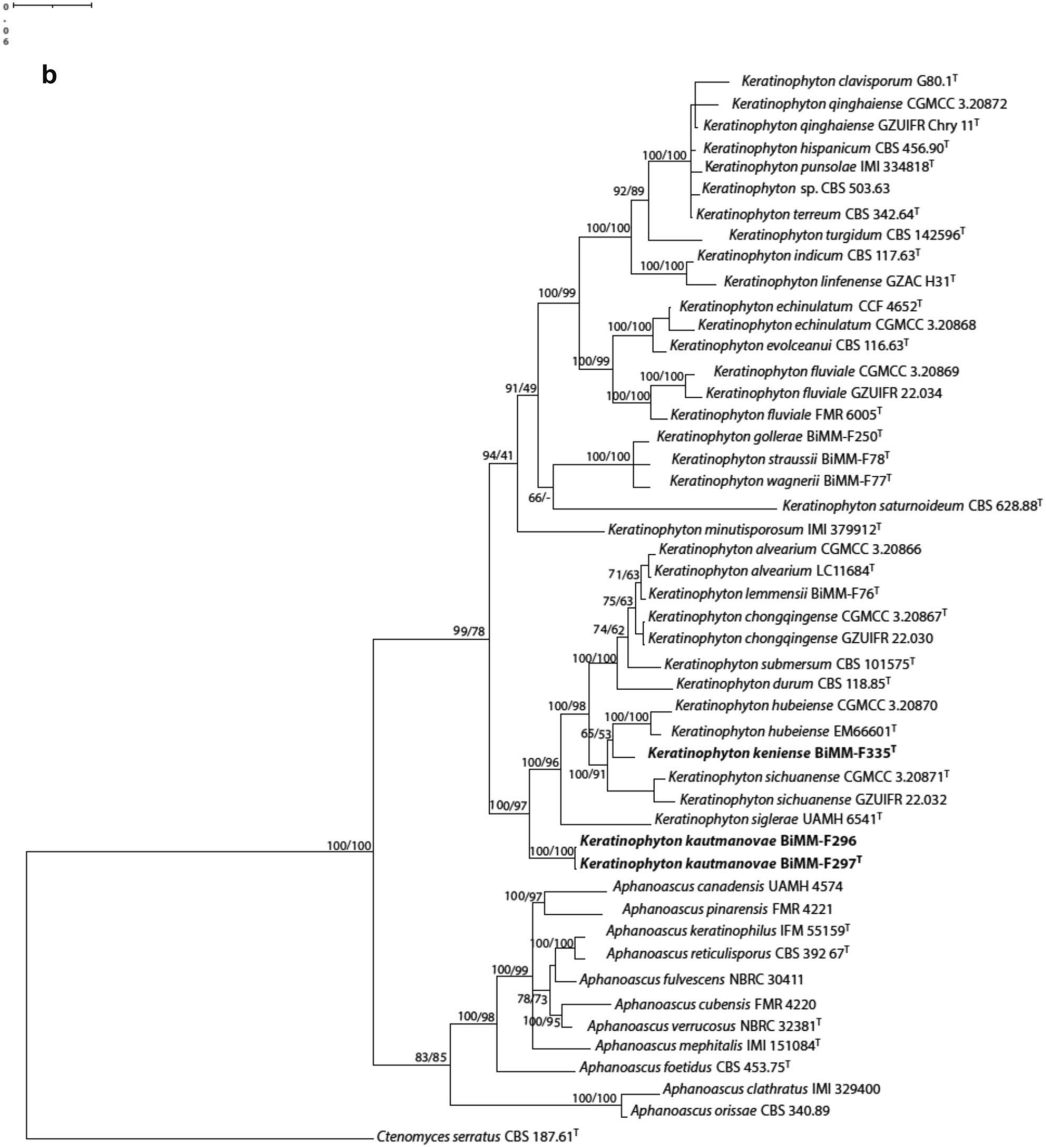
(**a**) Phylogenetic tree of *Keratinophyton* and *Aphanoascus* strains based on ITS sequences. The new taxa of *Keratinophyton* are compared with available sequences of the other related species together with their conidium size, presence of intercalary conidia and ability to grow at 37 °C. Empty triangles and squares represent the absence of the characteristics. (**b**) Phylogenetic tree based on combination of ITS and LSU sequences for the new taxa of *Keratinophyton* together with available sequences of the other related species. Numbers at nodes indicate Bayesian probability/ maximum likelihood bootstrap values (≥ 60%). New species are shown in bold. *Ctenomyces serratus* was used as outgroup. A sequence for *K*. *multiporum* was not available for the study. T, ex-type culture.

The ITS dataset consisted of 48 strains with 541 sites, LSU dataset consisted of 38 strains with 564 sites, and the ITS-LSU combined dataset included 38 strains with 564 sites. The best-fitting model was TNe + I + G4 for both datasets. Phylogenetic analyses of ITS (Fig. 1a) and combination of ITS-LSU data (Fig. 1b) of the species described in *Keratinophyton* and *Aphanoascus* revealed that strains BiMM-F297 and BiMM-296 (*Keratinophyton kautmanovae* sp. nov.) formed a basal clade in the genus *Keratinophyton* with 100% support. Strain BiMM-F335 (*Keratinophyton keniense* sp. nov.) was clustered together with *K. hubeiense* and *K. sichuanense* with 94% and 96% ITS similarity, respectively.

### Taxonomy

*Keratinophyton kautmanovae* B.Voleková, Kubátová, Kandemir & Labuda, sp. nov.

(Figs. 2 and 3)

**Figure 2:**
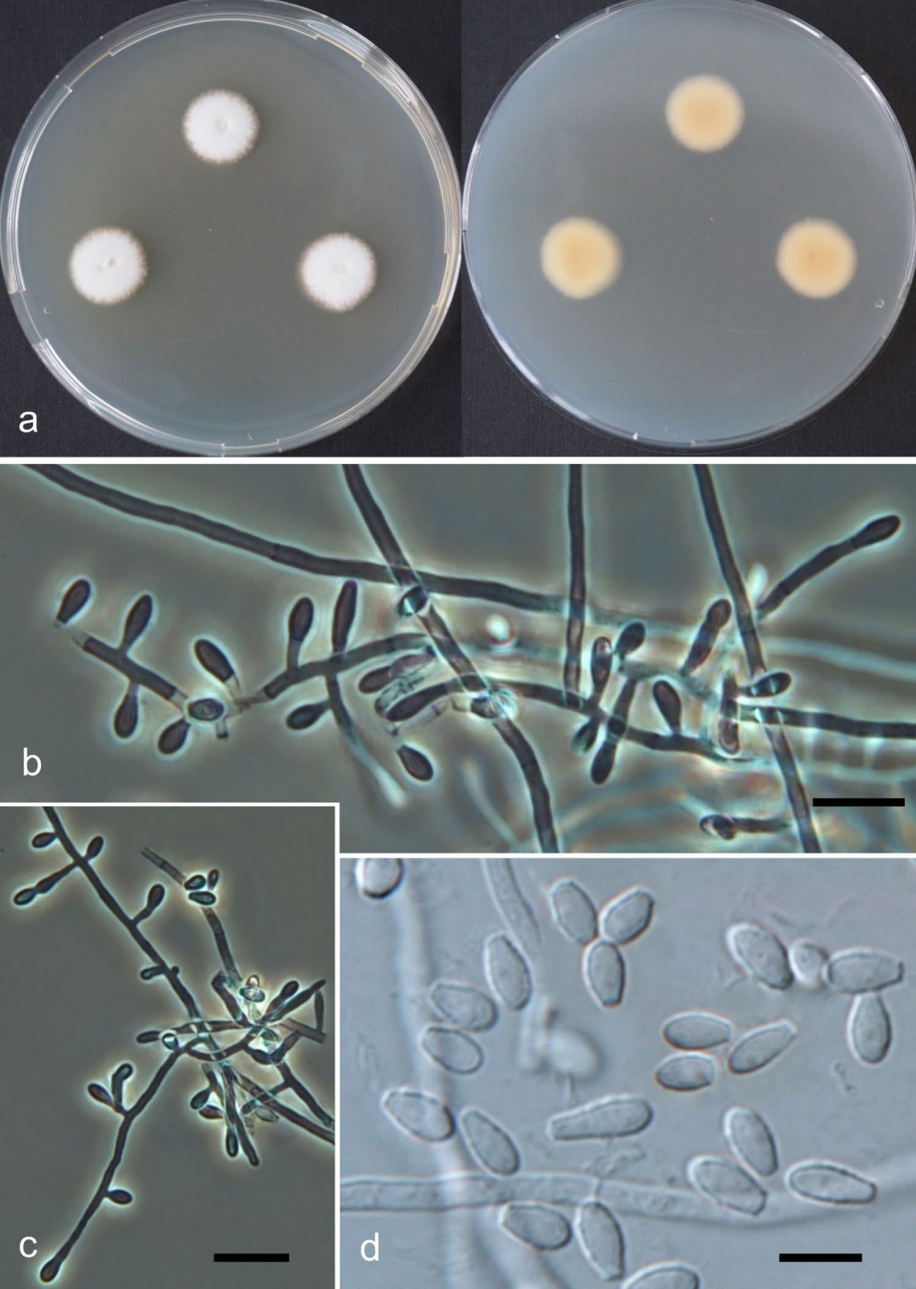
*Keratinophyton kautmanovae* (BiMM-F297). (**a**) Colonies on PDA (after 14 days) at 25 °C (left—obverse, right—reverse). (**b**,**c**) Conidiophores with aleurioconidia (phase contrast microscopy). (**d**) Aleurioconidia under light microscopy (on PDA, after 14 days). Bars = 10 µm (**b,c**), 5 µm (**d**).

**Figure 3:**
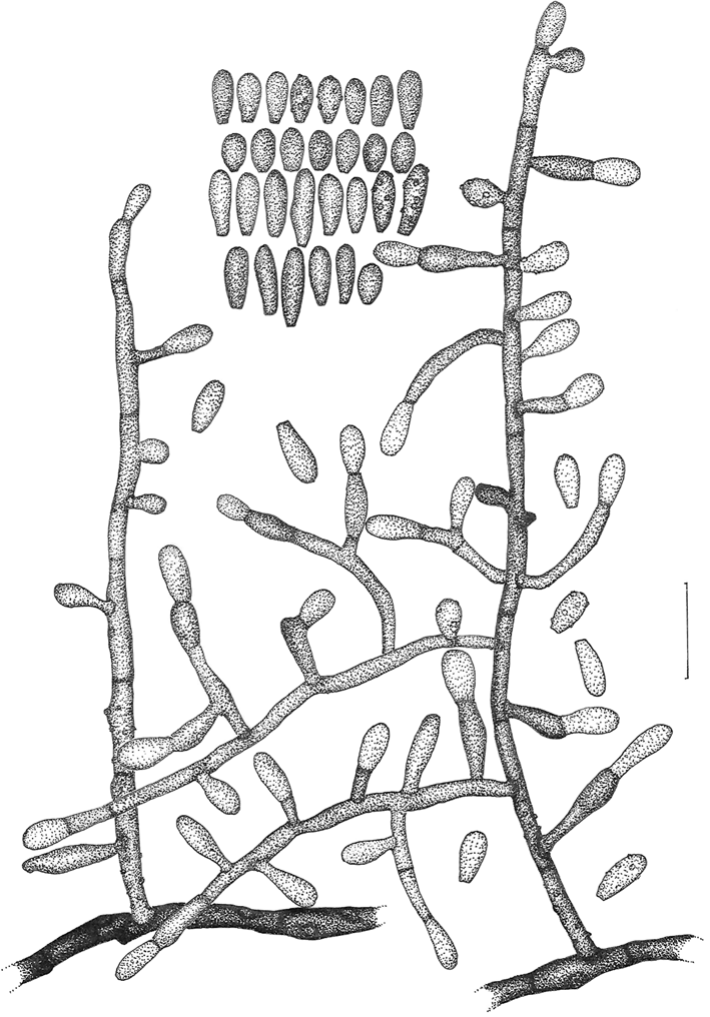
Line drawing of micromorphology of *Keratinophyton kautmanovae* (BiMM-F297). Conidiophores with young and mature aleurioconidia on PDA (at 25 °C, after 14 days). Branched conidiophore (at right) and unbranched conidiophore (at left) with sessile aleurioconidia. Fungus is not melanised. Bar = 10 µm.

*MycoBank*: MB851666

*Etymology:* Named in honour of Ivona Kautmanová, Department of Botany, Slovak National Museum-Natural History Museum, Bratislava, Slovak Republic, an expert in the fungal ecology and taxonomy of higher fungi.

*Type*: **Slovak Republic**, Malé Karpaty, Pezinok, Rudné Mines, (coordinates: 48°19′03.3"N 17°14′20.9"E), from a soil sample close (1 m) to stream of contaminated water of abandoned antimony Sb deposit, coll. B. Voleková, December 2021, isol. R. Labuda, February 2022, holotype PRM 957896 (dried culture in metabolically inactive state), culture ex-type BiMM-F297 = CCF 6679 = CBS 150893, ITS sequence, GenBank PP062810; LSU sequence, GenBank PP062954.

*Description*: *Sexual morph* not observed on any of the media used. *Asexual morph* on PDA (25 °C, 14 days, in dark). *Vegetative mycelium* of hyaline, septate, smooth-walled, sparsely to pronouncedly branched hyphae, 1.0‒2.0 µm diam. *Racquet hyphae* not observed. *Conidia* (aleurioconidia), hyaline, white in mass, thin-walled, mostly smooth to finely roughened, some also coarsely roughened or irregularly warty. Terminal and lateral conidia born on main fertile hyphae or from side branches of variable length, sessile or on short protrusions, often slightly swollen (ampuliform) and of variable length, solitary, 1‒3 (–5) per conidiogenous cell, smooth to verrucose (warty), thick-walled, obovate to clavate, 1-celled, (3.5‒)4.0‒5.0(‒6.5) × (2.0‒)2.0‒2.5(‒3.0) µm (mean = 4.5 ± 0.5 × 2.4 ± 0.2 µm, *n* = 50). *Intercalary conidia* (arthroconidia) not observed. *Chlamydospores* not observed.

*Culture characteristics*: *Colonies* on PDA 13‒15 mm diam at 25 °C, after 14 d, floccose to downy (mealy), with abundant sporulation, white to creamy, flat, slightly umbonate at the centre, with slightly radial colony margin submersed into agar, reverse yellowish with dark yellow centre, no pigment or exudate produced. At 30 °C, no growth (germination only). *Colonies* on SDA 7‒10 mm diam at 25 °C, after 14 days, morphology similar to when on PDA with more floccose and plane colony surface and radial colony margin, poor sporulation, with orange reverse. At 30 °C, no growth (no germination). *Colonies* on MEA 15‒20 mm diam at 25 °C, after 14 days, morphology similar to PDA with more floccose colonies and plane structure, with moderate sporulation, limited hyaline exudate present, with yellow-orange reverse and vivid orange centre. At 30 °C, no growth (germination only). No ascomata observed after prolonged incubation (3 months).

*Optimum temperature* for growth on PDA, SDA and MEA at 20–25 °C. *Minimum growth* (2–5 mm in diam) at 12 °C. Germination of the conidia observed at 10 °C. *Maximum temperature* for growth (1–3 mm in diam) at 28 °C. *Keratinolytic activity* very weak, with hair attack intensity = 0–1. *Urease activity* negative (after 20 days of incubation).

*Diagnosis: Keratinophyton kautmanovae* molecularly can be distinguished from other *Keratinophyton* species by ITS locus analysis. Combination of the following phenotypic features can be used to differentiate this fungus from other species in the genus: (1) obovoid-clavate, smooth to coarsely verrucose or irregularly warty conidia, (2) absence of arthroconidia, (3) generally slow grow at 25 °C, poor growth at 28 °C and no growth at 30 °C, and (4) orange reverse at 25 °C on MEA and SDA.

*Additional material examined*: Slovak Republic, Malé Karpaty, Pezinok, Rudné Mines, (coordinates: 48°19′03.3" N 17°14′20.9" E), isolated from a different sub-sample, February 2022, R. Labuda, a strain BiMM-F296 (ITS sequence, GenBank PP062811; LSU sequence, GenBank PP062955). Phenotypically and molecularly (ITS and LSU) identical with ex-type culture BiMM-F297.

*Notes:* Based on a search of NCBI GenBank nucleotide database, the closest hit for *Keratinophyton kautmanovae* using the ITS sequence is *Keratinophyton lemmensii* (ex—type CCF 6359 = BiMM—F 76; GenBank acc. MN633082), with identity = 463/504 (92%) and gaps 16/504 (3%). Phenotypically, *K. kautmanovae* can be readily distinguished from the *K. lemmensii* by absence of intercalary conidia (arthroconidia) and filiform 2-celled conidia, presence of orange colony reverse at 25 °C on MEA and SDA, slower growth at 25 °C and inability to grow at 30 °C.

*Keratinophyton keniense* V. Scheffenacker, A. Schüller, Kubátová, Kandemir & Labuda, sp. nov.

(Figs. 4 and 5)

**Figure 4:**
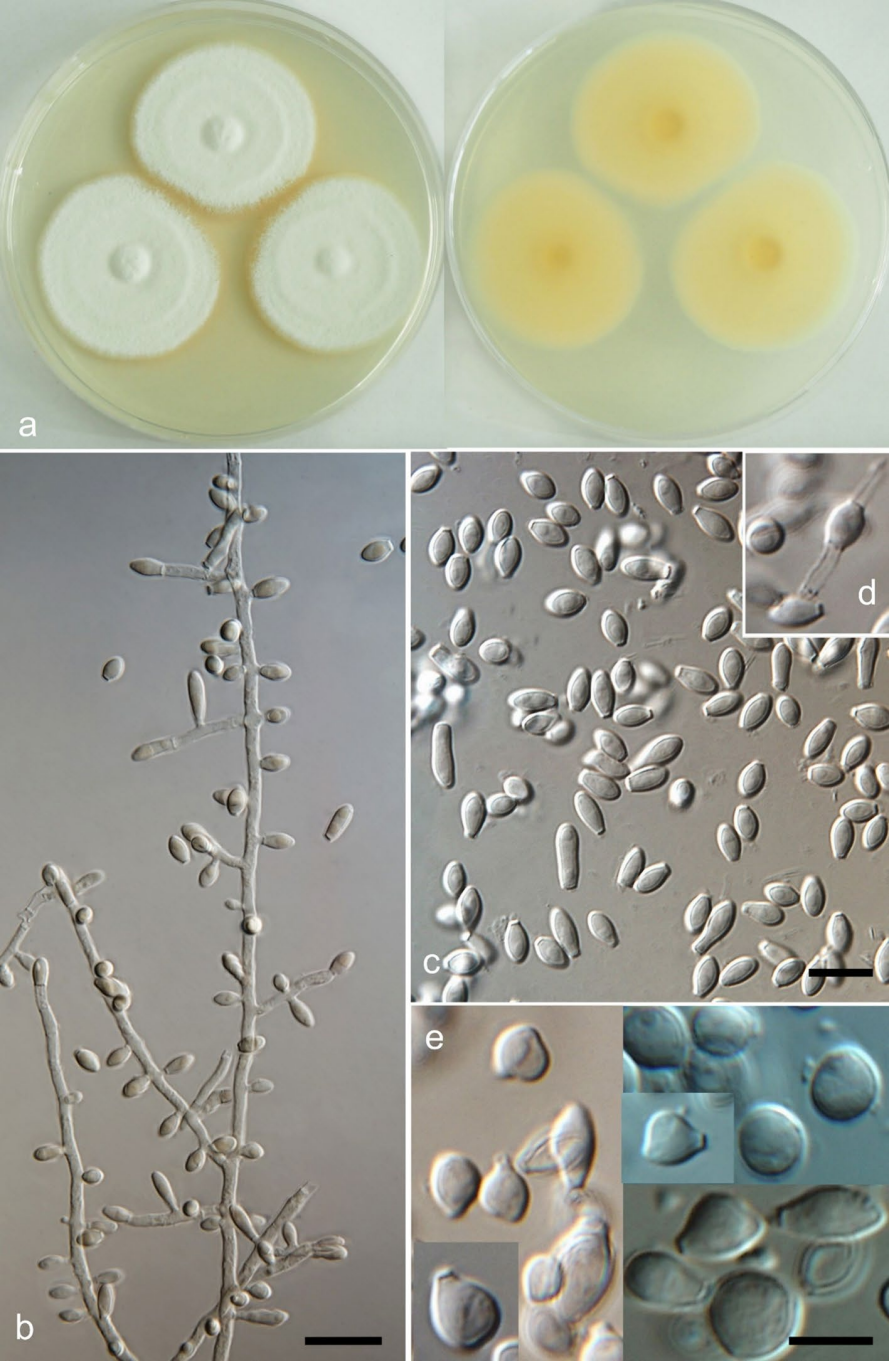
*Keratinophyton keniense* (BiMM-F335). **a** Colonies on PDA (after 14 days) at 25 °C (left—obverse, right—reverse). (**b**) Conidiophores with aleurioconidia. (**c–e**) Aleurioconidia. (**d**) Arthroconidium—intercalary conidium (on PDA, after 14 days). Bars = 10 µm.

**Figure 5:**
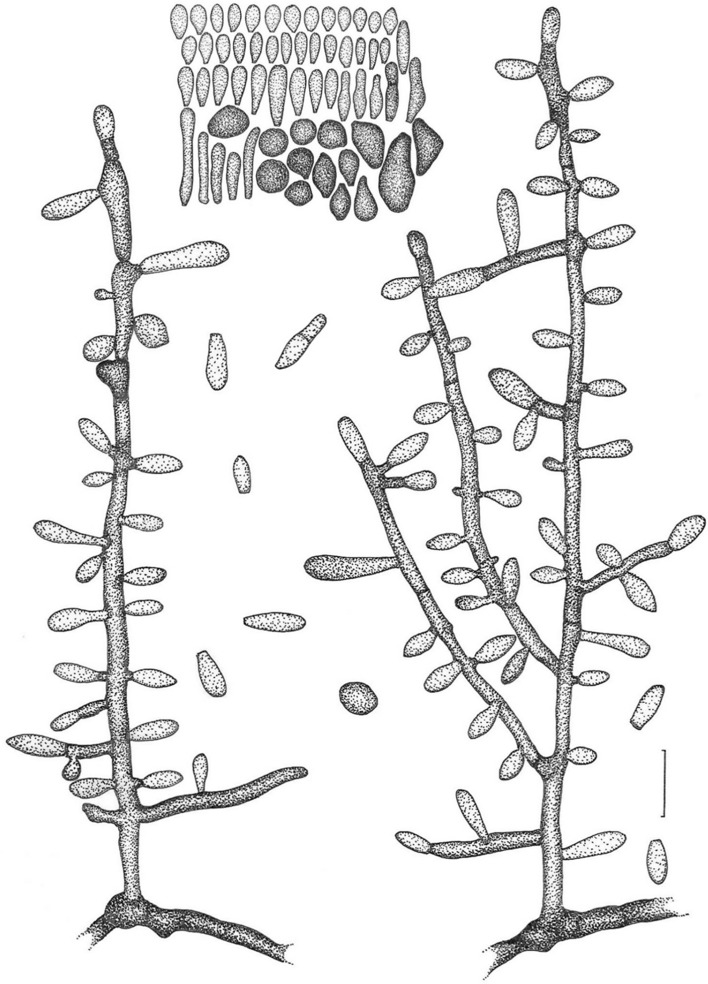
Line drawing of micromorphology of *Keratinophyton keniense* (BiMM-F335). Conidiophores with young and mature aleurioconidia on PDA (at 25 °C, after 14 days). Branched conidiophore (right) and unbranched conidiophore (left) with sessile aleurioconidia. Fungus is not melanised. Bar = 10 µm.

*MycoBank*: MB851667

*Etymology:* Named according to the country of origin, Kenya, where the holotype was collected.

*Type*: Republic of Kenya, Njoro, Egerton University campus (approximate coordinates: 0°22′09.3" S 35°55′24.2" E), from a soil sample (top layer, 20 cm), February 2022, coll. Andreas Schüller, isol. V. Scheffenacker, July 2023, holotype PRM 960013 (dried culture in metabolically inactive state), culture ex-type BiMM-F335 = CCF 6712 = CBS, ITS sequence, GenBank PP062809; LSU sequence, GenBank PP062953.

*Description*: *Sexual morph* not observed on any of the media used. *Asexual morph* on PDA (25 °C, 14 days, in dark). *Vegetative mycelium* of hyaline, septate, smooth-walled, sparsely to pronouncedly branched hyphae, 2.0‒5.0 µm diam. *Racquet hyphae* present, rare. *Conidia* (aleurioconidia) hyaline, white in mass, thick-walled, smooth to very finely roughened. Terminal and lateral conidia born on main fertile hyphae or from side branches of variable length, sessile or on short protrusions of variable length, solitary, 1‒3(–7) per conidiogenous cell, obovate to clavate, mostly 1-celled, smooth, thick-walled, often apiculate and cymbiform (boat-like shaped), (4.0‒)5.0‒6.0(‒7.0) × (2.0‒)2.5‒3.0(‒3.5) µm, (mean = 5.7 ± 0.8 × 2.8 ± 0.3 µm, *n* = 60), some also 2-celled, oblong to cylindrical, up to 18 µm long, conidia at colony centre often (sub-)globose and rhomboid, (4.5‒)5.0‒6.0(‒7.0) × (4.0‒)4.5‒5.5(‒6.0) µm (mean = 5.7 ± 0.4 × 4.7 ± 0.3 µm, *n* = 60). *Intercalary conidia* (arthroconidia) very rare. *Chlamydospores* not observed.

*Culture characteristics*: *Colonies* on PDA 35‒38 mm diam at 25 °C, after 14 days, floccose to downy (mealy), with abundant sporulation, white to creamy, flat, slightly umbonate at the centre with a few concentric rings, even regular colony margin submersed into agar, reverse yellow with dull yellow centre, no pigment or exudate produced. At 30 °C, 33‒37 mm diam. *Colonies* on SDA 37‒40 mm diam at 25 °C, after 14 days, morphology similar to when on PDA, good sporulation, with darker yellow reverse at the centre. At 30 °C, 35‒38 mm diam. *Colonies* on MEA 43‒45 mm diam at 25 °C, after 14 days, powdery to downy (mealy), with abundant sporulation, white to creamy, flat, slightly umbonate at the centre, margin irregular, reverse yellow to dull yellow orange, no pigment or exudate produced. At 30 °C, 29‒31 mm diam. No ascomata observed after prolonged incubation (3 months).

*Optimum temperature* for growth on PDA, SDA and MEA at 20–30 °C. *Minimum growth* (2–6 mm in diam) at 10 °C. Germination of the conidia observed at 8 °C. *Maximum temperature* for growth (2–5 mm in diam) at 33 °C. Germination of the conidia and formation of microcolonies observed at 34 °C. *Keratinolytic activity* absent, with hair attack intensity = 0. *Urease activity* negative (after 20 days of incubation).

*Diagnosis: Keratinophyton keniense* molecularly can be distinguished from other *Keratinophyton* species by ITS locus analysis. Combination of the following phenotypic features can be used to differentiate this fungus from other species in the genus: (1) cymbiform and rhomboid conidia, (2) arthroconidia very rare, (3) good grow at 20–30 °C, (4) dull yellow reverse at 25 °C.

*Notes:* Based on a search of NCBI GenBank nucleotide database, the closest hit for *Keratinophyton keniense* using the ITS sequence is *Keratinophyton sichuanense* (ex—type CGMCC 3.20871; GenBank acc. NR182583), with identity = 529/550 (96%) and gaps 1/550 (0%). Phenotypically, *K. keniense* can be readily distinguished from the *K. sichuanense* by presence of rhomboid conidia, slower grow on PDA at 25 °C after 14 days (35‒38 vs 50‒54 mm), and inability to grow at 37 °C.

## Discussion

### Phylogeny

Phylogenetic reconstruction using ITS and LSU sequences (Fig. 1) resulted in clustering the both new taxa, *Keratinophyton kautmanovae* and* K. keniense*, with eight currently accepted species, namely *K. alvearium*^14^, *K. durum*^2^*, K. chongqingense*^8^, *K. hubeiense*^12^, *K. lemmensii*^7^, *K. sichuanense*^8^, *K. siglerae*^29^, and *K. submersum*^11^. Apart from the pronounced differences in the ITS regions, the species mentioned above can be distinguished by particular combinations of their phenotypic traits (e.g., colony characteristics, and morphology of conidia) as listed in Table 2. The monophyletic genus *Keratinophyton* is now extended and includes 29 species including six species known from sexual morphs^5^ and 23 species which are currently known only from asexual morphs^8^ and this study. The ability to produce ascosporic state (sexual morph) in vitro within this cluster is confined to *K. durum*^2^, characterized by discoid ascospores with flattened poles and with a broad equatorial rim, cruciform in side view, broad-ridged, with reticulate surface^4^. As noted by Li et al. (2022)^8^, the ability to form sexual morph in vitro is not phylogenetically conserved, as it can be seen from the phylogenetic analysis, showing that all known species forming ascosporic (sexual) structures within the genus are not clustered together and they are spread over the phylogenetic tree^6–8^.

**Table 2:** Comparison of the key phenotypic characteristics of the phylogenetically close related *Keratinophyton* spp.

Species	Reverse colony on PDA 25 °C, after 14 days	Intercalary conidia	Conidial shape	Conidial dimensions (µm)	Conidial surface	References
***K. kautmanovae***	Yellowish, dark yellow centrally	Absent	Obovate to clavate	3.5‒6.5 × 2.0‒3.0 (1-celled)	Smooth to verrucose	**This study**
***K. keniense***	Yellowish, dull yellow centrally	Present^b^	Obovate to clavate, cymbiform, filiform, globose, rhomboid	4.0‒18.0 × 2.0‒6.0 (1- to 2-celled)	Smooth	**This study**
*K. alvearium*	White	Present^c^	Globose, pyriform, clavate, cylindrical	4.0‒9.0 × 2.0‒7.5 (1-celled)	Smooth	Zhao et al., 2018^14^
*K. chongqingense*	White	Present	Subglobose, globose, obovate, ellipsoidal apiculate	3.0‒10.0 × 2.0‒4.5 (1-celled)	Smooth to slightly verrucose	Li et al., 2022^8^
*K. durum*^a^	Colorless	Absent	Pyriform to clavate	4.5‒7.7 × 2.0‒3.0 (1-celled)	Smooth	Guarro et al. 2012^4^
*K. hubeiense*	Yellowish	Absent	Obovoid to ellipsoidal	2.0‒4.5 × 1.5‒3.0 (1-celled)	Smooth	Zhang et al. 2016^43^
*K. lemmensii*	Lemon yellow (with bright yellow pigment)	Present	Clavate to filiform	3.0‒40.0 × 1.5‒4.0 (1- to 2-celled)	Smooth	Labuda et al., 2021^7^
*K. sichuanense*	White	Absent	Obovate to clavate, sometimes curved	4.0‒8.5 × 2.0‒4.0 (1-celled)	Smooth to slightly verrucose	Li et al., 2022^8^
*K. siglerae*	Pale brown	Present	Cylindrical to clavate	5.0‒30.0 × 2.0‒3.5 (1- to 2-celled)	Smooth to slightly verrucose	Cano et Guarro, 1994^29^
*K. submersum*	Yellowish white	Present^d^	Clavate, pyriform, obovoid and subglobose	4.0‒35.0 × 2.5‒5.0 (1- to 4-celled)	Smooth to verrucose	Vidal et al., 2002^11^

^a^Forming sexual morph (ascospores) in vitro.

^b^Intercalary conidia observed only very rarely.

^c^Intercalary conidia abundant, solitary or in chains.

^d^Intercalary conidia present in older cultures.

### Ecology and distribution

Almost all known *Keratinophyton* species have been isolated from soil or soil-like substrates, such as river sediments, compost and sand, and as non-pathogenic fungi as a result of mycological screening for so-called keratinophilic/keratinolytic fungi using a horse-hair baiting method^7,8^. This highly selective method was introduced by Vanbreuseghem (1952) for soil fungi having affinity to keratinous material especially for onygenalean fungi such as dermatophytes^19,30^. According to Papini et al. (1998), Ajello reviewed the taxonomy of keratinophilic fungi for the first time in 1968^31^. Later, Otčenášek et al. (1969) reported on the worldwide distribution of keratinophilic mycobiota in soil, claiming that the occurrence of keratinophilic fungi in soil depends on the presence of mammals, birds, and humans in a variety of ecological sites^32^. It is in fact the only method how this group of keratinophilic fungi can be isolated from the soil-like substrates and studied further. In this study, both fungi originated from the areas which are freely accessible by wild animals typically inhabiting these regions, and thus, these soils might be presumably reach on source of keratin as well as a source of biodiversity for this specific fungal group regardless of geochemical properties of soils^15,33,34^. As for the sample from the abandoned antimony (Sb) deposit (Slovakia, EU), the soil sample is rich in iron oxides and is also characterized by elevated concentrations of arsenic, antimony, aluminum and sulfates. More about mineralogy and geochemistry of the studied site was published previously^35^. On the other hand, the soils in Egerton Njoro area are Vintric Mollic andosols^36,37^. The sampled area is native, not influenced by agricultural or any industrial activities.

As mentioned above, the genus *Keratinophyton* harbours a total of 29 species, including the two new species described in this study. The majority of the new taxa have been so far described from Europe (15 spp.), followed by Asian continent (China, 8 spp. and India, 5 spp.)^5–8^. To the best to our knowledge, the description of *K. keniense* represents the first taxonomical novelty of the genus *Keratinophyton* from African continent. Thus, further research is needed because unknown strains may be isolated from similar environments on the African continent.

Hubálek provided a list of keratinolytic fungi associated with free-living mammals and birds of which ubiquitous *K. durum*, *K. pannicola* and *K. terreum* have been isolated from a variety of animals and from different geographical regions^38^. There are only a few reports of a human or animal clinical isolate belonging to *Keratinophyton*^16,39^, however, all these cases have doubtful etiological history and with no solid evidence of their pathogenicity^16,40^. On the other side, *K*. *pannicola* (as *C*. *pannicola*) is included in the Atlas of Clinical Fungi^17^ as a concern in skin infections. Even though the keratinophilic fungi were considered as potential pathogens by several researchers^41,42^; they rarely cause infections. Therefore, soil is proposed as an epidemiological and probably also an evolutionary link, that relates geophilic, zoophilic, and anthropophilic keratinophilic fungi^42^. Although being cycloheximide resistant, a potential pathogenicity to homeothermic vertebrates (mammals and birds) by these fungi seems highly unlikely because both new species are not able to grow at higher temperature (above 30‒34 °C), they are urease negative, and possess none or very mild keratinolytic activity in vitro. Rather contrary, these fungi might be interesting from a metabolic point of view, as they undoubtedly represent a yet unexplored source of new bioactive compounds as there is not much known of these properties in the genus^34^. Metabolic profile and investigation of the potential use of substances produced by these two novel fungi is an object of our further biochemical exploration.

### Supplementary Information

Supplementary Table S1.

## Data Availability

The phylogenetic trees constructed for the study can be found in TreeBASE, http://purl.org/phylo/treebase/phylows/study/TB2:S31056. The data analysed in this study are also available from the corresponding author on reasonable request.
