# Supplementary material for: Two novel members of Onygenales, Keratinophyton kautmanovae and K. keniense spp. nov. from soil
Source: Sci Rep. 2024 Jul 17;14:16525. doi: 10.1038/s41598-024-67475-y (PMC11254920; doi:10.1038/s41598-024-67475-y)
Supplement: Supplementary file 1 — Supplementary Table S1. [file 41598_2024_67475_MOESM1_ESM.docx]

**Supplementary information**

**Table S1a** Temperature dependent growth (in mm colony diameter) of the new *Keratinophyton* species on **PDA** after 14 d.

| Species | Temperature (°C) | | | | | | | | | | | |
| --- | --- | --- | --- | --- | --- | --- | --- | --- | --- | --- | --- | --- |
|  | **8** | **10** | **12** | **15** | **20** | **25** | **28** | **30** | **32** | **33** | **34** | **35** |
| *K. kautmanovae* | nSG | SG | 2‒3 | 5‒10 | 10‒12 | 13‒15 | 1‒2 | G | nSG | nSG | - | - |
| *K. keniense* | SG-MC | 3‒4 | - | 11‒14 | 32‒35 | 35‒38 | - | 40‒43 | 18‒20 | 3‒4 | SG-MC | nSG |

SG, spore germination; nSG, no spore germination; MC, microcolonies;

**Table S1b** Temperature dependent growth (in mm colony diameter) of the new *Keratinophyton* species on **MEA** after 14 d.

| Species | Temperature (°C) | | | | | | | | | | | |
| --- | --- | --- | --- | --- | --- | --- | --- | --- | --- | --- | --- | --- |
|  | **8** | **10** | **12** | **15** | **20** | **25** | **28** | **30** | **32** | **33** | **34** | **35** |
| *K. kautmanovae* | nSG | SG | 2‒5 | 7‒10 | 12‒15 | 15‒20 | 1‒2 | SG | nSG | nSG | - | - |
| *K. keniense* | SG-MC | 2‒3 | - | 10‒13 | 24‒30 | 43‒45 | - | 28‒35 | 10‒14 | 2‒5 | SG | nSG |

SG, spore germination; nSG, no spore germination; MC, microcolonies

**Table S1c** Temperature dependent growth (in mm colony diameter) of the new *Keratinophyton* species on **SDA** after 14 d.

| Species | Temperature (°C) | | | | | | | | | | | |
| --- | --- | --- | --- | --- | --- | --- | --- | --- | --- | --- | --- | --- |
|  | **8** | **10** | **12** | **15** | **20** | **25** | **28** | **30** | **32** | **33** | **34** | **35** |
| *K. kautmanovae* | nSG | SG | 1‒2 | 5‒6 | 6‒9 | 7‒10 | M‒1 | nSG | nSG | - | - |  |
| *K. keniense* | SG-MC | 4‒5 | - | 7‒9 | 23‒25 | 37‒40 | - | 32‒37 | 18‒19 | 1‒2 | MC | nSG |

SG, spore germination; nSG, no spore germination; MC, microcolonies
